# Supplementary material for: BcABF1 Plays a Role in the Feedback Regulation of Abscisic Acid Signaling via the Direct Activation of BcPYL4 Expression in Pakchoi
Source: Int J Mol Sci. 2024 Mar 30;25(7):3877. doi: 10.3390/ijms25073877 (PMC11011251; doi:10.3390/ijms25073877)
Supplement: Supplementary file 1 [file ijms-25-03877-s001.zip › ijms-2882031-supplementary.pdf]

Supporting information:

Figure S1: The phenotypic indicators of *BcABF1* transgenic plants.

Figure S2: Interaction analysis of *BcABF1* and *BcPYLs*.

Table S1: Primers used in this study.

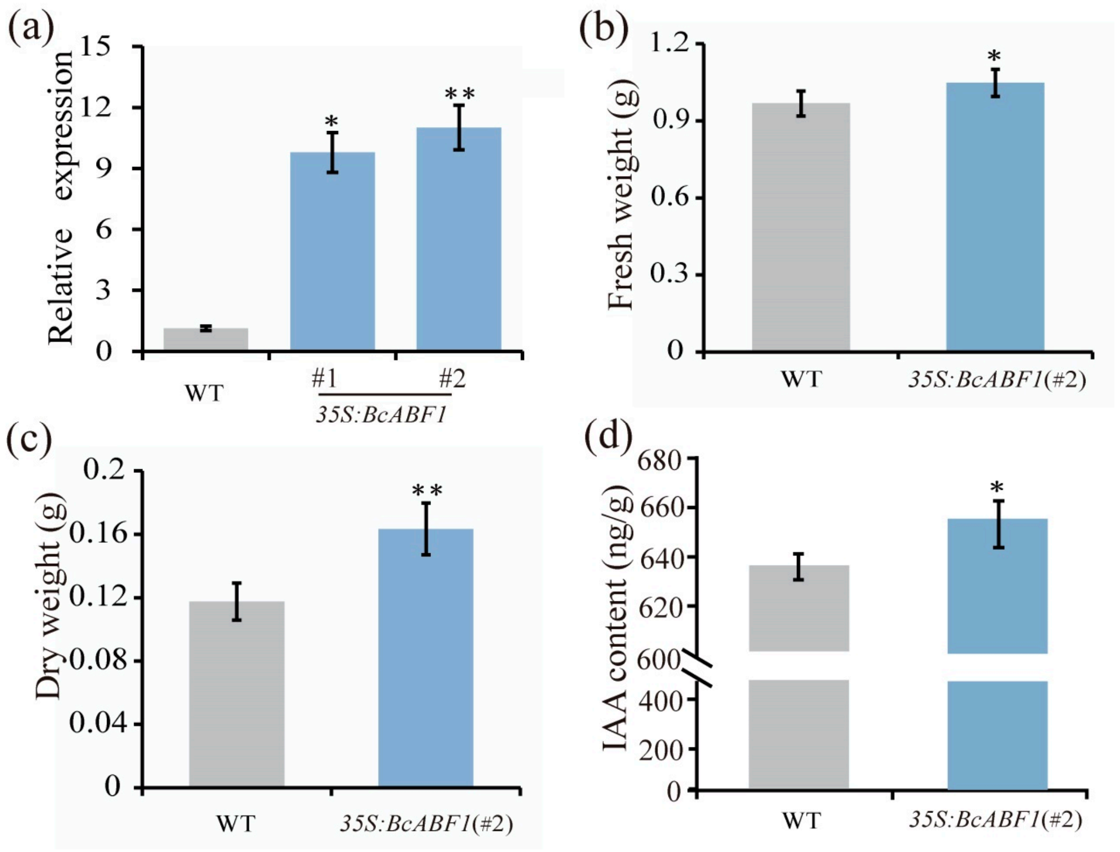

Figure S1: The phenotypic indicators of *BcABF1* transgenic plants.
(a)The mRNA abundance of *BcABF1* in *35S:BcABF1*. (b) Fresh weight. 28-day *35S:BcABF1* and WT were used to measure fresh weight (c) Dry weight. 28-day *35S:BcABF1* and WT were used to measure dry weight. (d) IAA content in *35S:BcABF1*. Data are three biological repeat averages (***p* < 0.01, **p*<0.05, Student's *t*-test).

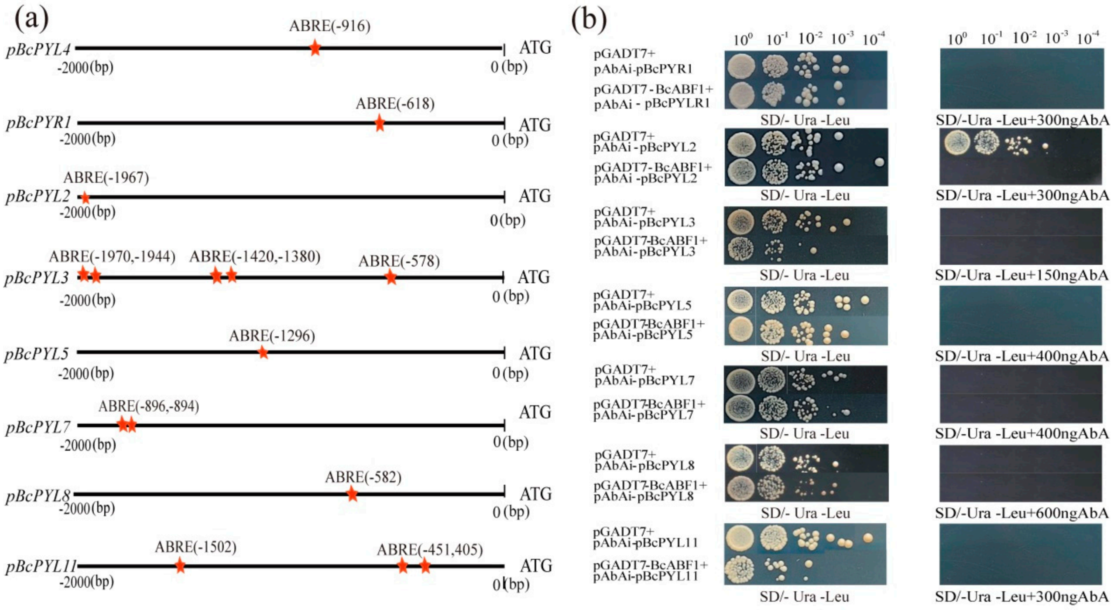

Figure S2: Interaction analysis of *BcABF1* and *BcPYLs.*

(a) *BcPYLs* promoter cis-element analysis. (b) Y1H analysis of *BcABF1* and *BcPYLs*.

Table S1: Primers used in this study

Primer Name	Sequences (5'-3')	Function
BcABF1-F	ATGGGTACTCACATCAATTT	Cloning
BcABF1-R	TTACCATGGACCGGTTAAGG	
BcPYL4-F	ATGCTCGCCGTACACCGCCCTTCCTCCG	
BcPYL4-R	CATGGACGTCTTCTTCTTCCCTTCCACC	
pBcPYL4-F	GTGGTTGAGAACCTGAATGGAT	
pBcPYL4-R	GTTCTTGTGTTTTATAGAA	
pBcPYR1-F	ATCAAGCAGGGAGAGCTATGGAAACG	
pBcPYR1-R	GAGATGTATTTATGTGGGACACAGGs	
pBcPYL2-F	CCAATGCCTTCTTCAATCA	
pBcPYL2-R	GATGATGAATAGATCACACGTG	
pBcPYL3-F	ATTTATAATTTTTTTAAAGATATGAAAGTTA	
pBcPYL3-R	CTCCATATGGTACGTGCTGGTG	
pBcPYL5-F	TGACGGCCGCGGCTGTGTTCTC	
pBcPYL5-R	TCTTGTCTTTATAGTTTTATTAATTGCGATTAT	
pBcPYL7-F	AAAGTCAGACTACTCTTCTCC	
pBcPYL7-R	AAATTAGCTTTTATAATATTTTTTCTTTTTAT	
pBcPYL8-F	CTTGCTCAATATAATGGTAAGTCT	
pBcPYL8-R	CTTAATTTTATTTTTCTACCTTTTTTTTG	
pBcPYL11-F	TAAGAAAAAATGAACTGATGATTTGTTGG	
pBcPYL11-R	TTGTTTGCTTTTTTACGCTTGTGCCC	
pRI101-BcABF1-F	TCTTCACTGTTGATACATATGATGGGTACTCACATCAATTTCAACA	Dual-luciferasreporter assay
pRI101-BcABF1-R	GCTCACCATGGATCCGGTACCCCATGGACCGGTTAAG	
p0800-pBcPYL4-F	CTTGATATCGAATTCCTGCAGGTGGTTGAGAACCTGA	
p0800-pBcPYL4-R	CGCTCTAGAACTAGTGGATCCGTTCTTGTGTTTTATA	
pAbAi-pBcPYL4-F	CTTGAATTCGAGCTCGGTACCGTGGTTGAGAACCTGA	Yeast-one-hybrid assay
pAbAi-pBcPYL4-R	ATACAGAGCACATGCCTCGAGGTTCTTGTGTTTTATA	
AD-BcABF1-F	GCCATGGAGGCCAGTGAATTCATGGGTACTCACATC	
AD-BcABF1-R	CAGCTCGAGCTCGATGGATCCCCATGGACCGGTTAA	
pAbAi-pBcPYR1-F	CTTGAATTCGAGCTCGGTACCATCAAGCAGGGAGAGC	
pAbAi-pBcPYR1-R	ATACAGAGCACATGCCTCGAGGAGATGTATTTATGTGGG	
pAbAi-pBcPYL2-F	CTTGATATCGAATTCCTGCAGCCAATGCCTTCTTCA	
pAbAi-pBcPYL2-R	ATACAGAGCACATGCCTCGAGGATGATGAATAGATCA	
pAbAi-pBcPYL3-F	CTTGATATCGAATTCCTGCAGATTTATAATTTTTTTAAAGAT	
pAbAi-pBcPYL3-R	ATACAGAGCACATGCCTCGAGCTCCATATGGTACGT	
pAbAi-pBcPYL5-F	CTTGATATCGAATTCCTGCAGTGACGGCCGCGGCTG	
pAbAi-pBcPYL5-R	ATACAGAGCACATGCCTCGAGTCTTGTCTTTATAGTTTTA	
pAbAi-pBcPYL7-F	CTTGATATCGAATTCCTGCAGAAAGTCAGACTACTCT	
pAbAi-pBcPYL7-R	ATACAGAGCACATGCCTCGAGAAATTAGCTTTTATAATAT	
pAbAi-pBcPYL8-F	CTTGATATCGAATTCCTGCAGCTTGCTCAATATAATGG	
pAbAi-pBcPYL8-R	ATACAGAGCACATGCCTCGAGCTTAATTTTATTTTTCT	
pAbAi-pBcPYL11-F	CTTGATATCGAATTCCTGCAGTAAGAAAAAATGAACTGA	
pAbAi-pBcPYL11-R	ATACAGAGCACATGCCTCGAGTTGTTTGCTTTTTTACGC	

PBI121-pBcPYL4-F<br>PBI121-pBcPYL4-R	ACCATGATTACGCCAAGCTTGTGGTTGAGAACCTGA<br>GACTGACCACCCGGGGATCCGTTCTTGTGTTTTATA	**β-Glucuronidase staining and expression**
AtPYL4-Q-F	ATATCCTCGACGACGAACGC	**qRT-PCR**
AtPYL4-Q-R	CACCACCAACAACGCTGAAG	
PP2A-Q-F	AGGCTACACGTTCGGACAAG	
PP2A-Q-R	TGGGGCACTAAACACAGTCA	
EIFA-Q-F	TGACCACACAGTCTCTGCAA	
EIFA-Q-R	ACCAGGGAGACTTGTTGGAC	
BcABF1-Q-F	TCCGAGCTGGTGTTGTGAAA	
BcABF1-Q-R	GCATTGTTGCTGGTGCTTGA	
BcPYL4-Q-F	AGTTTCAGCGTCGTTGGTGG	
BcPYL4-Q-R	CAACAACCACGGTTCCACAGA	
CYS5-Q-F	GGTTGGAGTCCCATCAGCAA	
CYS5-Q-R	CCGTCTCGAACTTGAGTCCC	
RD29A-Q-F	TGCACCGGCTCATTCTGTAA	
RD29A-Q-R	GCAGAGAGACCGGAGTGTTC	
RD29B-Q-F	ACCATCCAGAAGAAGAAGAGCA	
RD29B-Q-R	TCAACACTTTGGATGCTCCC	
RAB18-Q-F	AGCTCGGAGGATGATGGACA	
RAB18-Q-R	AGCCACCAGCATCATATCCG	
KIN2-Q-F	GGAGCTTCCGCGCAACAG	
KIN2-Q-R	GTTAACACCTCCCACTGCCG	
SOC1-Q-F	GCTCAAGCAAAAGGAGAAAGC	
SOC1-Q-R	TCGCTTTCATGAGATCCCCAC	
NYE1-Q-F	TACCGGGATGAAGTTGTGGC	
NYE1-Q-R	CCGTCTCGAACTTGAGTCCC	
NYC1-Q-F	TCATTATCGAGCCGGTCCAC	
NYC1-Q-R	TCCCCTAGTGCTTCCGGTTA	

Note: The underline represents the enzyme cutting site.
